# Elucidating the polycyclic aromatic hydrocarbons involved in soot inception

**DOI:** 10.1038/s42004-023-01017-x

**Published:** 2023-10-16

**Authors:** Can Shao, Qi Wang, Wen Zhang, Anthony Bennett, Yang Li, Junjun Guo, Hong G. Im, William L. Roberts, Angela Violi, S. Mani Sarathy

**Affiliations:** 1 King Abdullah University of Science and Technology (KAUST), Clean Combustion Research Center, Thuwal, 23955-6900 Saudi Arabia; 2 Department of Mechanical Engineering, University of Michigan, 2350 Hayward St, Ann Arbor, MI 48109-2125 USA; 3 King Abdullah University of Science and Technology (KAUST), Core Labs, Thuwal, 23955-6900 Saudi Arabia; 4 Science and Technology on Combustion, Internal Flow and Thermostructure Laboratory, School of Astronautics, Northwestern Polytechnical University, 710072 Xi’an, China; 5 Department of Chemical Engineering, University of Michigan, 2350 Hayward St, Ann Arbor, MI 48109-2125 USA; 6 Chemical Engineering and Biophysics Program, University of Michigan, 930 N. University Ave, Ann Arbor, MI 48109-1055 USA

**Keywords:** Reaction kinetics and dynamics, Carbohydrate chemistry, Fossil fuels, Reaction mechanisms

## Abstract

Polycyclic aromatic hydrocarbons are the main precursors to soot particles in combustion systems. A lack of direct experimental evidence has led to controversial theoretical explanations for the transition from gas-phase species to organic soot clusters. This work focuses on sampling infant soot particles from well-defined flames followed by analysis using state-of-the-art mass spectrometry. We found that PAH molecules present in soot particles are all stabilomers. Kinetic Monte Carlo simulations and thermodynamic stability calculations further identify the detected PAHs as peri-condensed and without aliphatic chains. Van der Waals forces can easily link PAHs of such size and shape to form PAH dimers and larger clusters under the specified flame conditions. Our results provide direct experimental evidence that soot inception is initiated by a physical process under typical flame conditions. This work improves our understanding of aerosol particulates, which has implications for their environmental and climate change impacts.

## Abstract (web summary)

Polycyclic aromatic hydrocarbons (PAHs) are the main precursors to soot particles in combustion systems, but the transition from gas-phase species to organic soot clusters is still not fully understood. Here, the authors study infant soot particles with mass spectrometry and kinetic Monte Carlo simulations and identify peri-condensed PAHs to be the most thermodynamically stable species.

## Introduction

Soot and its precursors, polycyclic aromatic hydrocarbons (PAHs), are formed from the incomplete combustion of hydrocarbon fuels^1,2^. The formation of such particles has broad implications on the environment^3,4^, human health^5^, material synthesis^6^, and the formation of interstellar carbonaceous particles^7,8^.

Extensive studies have focused on the formation pathways of PAHs^6,9–11^ and surface reactions of soot particles^11,12^, but the transition from gas-phase PAHs to nascent soot largely remains a mystery. This transition, called inception, is a highly selective process, during which numerous PAHs remain in the gas phase and only a fraction of them participated in inception. Three predominant hypotheses explain such selectivity for the inception mechanism: (1) the combination of two-dimensional PAHs with fullerene-like structures^13^, (2) the physical coalescence of PAHs into stacked clusters^14,15^, and (3) the chemical coalescence of PAHs into cross-linked, three-dimensional structures^16,17^. However, little direct evidence supports any of these hypotheses.

Indirect experimental and computational studies provide insight into the inception process. The first pathway is too slow to explain the soot inception rate observed in the hydrogen-abstraction/acetylene (C_2_H_2_) addition framework^11,18,19^, which is the dominant mechanism for PAH growth under many combustion conditions. Soot particles generated using this pathway have a C/H ratio of around 10, much higher than those measured in existing experiments^20^. Therefore, most recent theoretical calculations on soot inception focus on the latter two pathways^14,16,21–23^. In addition, new insight into infant soot inception processes has been gained through advanced diagnostic techniques.

In 1991, D’Alessio et al. discovered the presence of nano-organic carbon (NOC) particles in premixed flames^24^. At the same time, Dobbins et al.^25^ reported that small polydisperse singlet particles were detected at intermediate temperatures on the fuel side of a diffusion flame. Later, D’Anna et al.^26^ used ultraviolet–visible spectroscopy and laser scattering/extinction techniques for soot particle volume fraction and size measurement in diffusion flames. Their study identified two types of particles: NOC, which are transparent to visible radiation, and solid black soot particles that absorb light across the whole spectral range^26^. The NOC particles can reach concentrations comparable to mature soot particles and are usually formed early in the flame^26,27^. Kholghy et al.^28^ found that NOC particles are liquid-like and form at temperatures below 1500 K. When the temperature exceeds 1500 K, the transition of the transparent particles into mature agglomerated particles occurs instantly. Cain et al.^20^ reported that the liquid-like particles comprise peri-condensed PAH in the mass range of 200 to 600 amu. In addition, Dobbins et al.^29–31^ made similar observations and further demonstrated that the transition from the precursor to mature particles is accomplished by carbonization^30^. For premixed flame and diffusion flames, the chemical environments for particle formation are very different due to the H-enriched environment in the former and the H-diminished environment in the latter. When H atoms are abundant, aryl radicals can be generated by H-abstraction reactions, which results in enhancing the chemical nucleation pathway^6^. However, in the post flame zone of premixed flame or in diffusion flames, H-atom concentrations are too low to initiate such reactions.

Studying the inception process is still extremely difficult despite these advances in experimental techniques. Several obstacles hinder experimental research on soot inception. First, the process from the gas phase to particles is transitory, and it is challenging to pinpoint the actual transition point^20,32–35^. In addition, particles generated during and post sampling may cause significant perturbation to the experimental results. For example, Adamson et al.^35,36^ detected pyrene dimers in an ethylene nonpremixed flame using a flame-sampling tandem mass spectrometer. However, these were generated in the sampling line. The present work provides further experimental evidence that pyrene dimers cannot be a key intermediate in particle inception at elevated flame temperatures^35^.

This study provides direct evidence elucidating the inception pathway by analyzing NOC particles (or infant soot) using a combination of experimental and computational methods. A burner-stabilized stagnation flame technique was used, coupled with micro-orifice probe sampling^37^ to accumulate NOC particles onto a copper grid surface. This method minimizes secondary collisions of sampled particles and PAHs. In addition, NOC particles were also sampled from a co-flow nonpremixed flame through traditional thermophoresis sampling. The NOC particles generated in both premixed and nonpremixed flames were obtained in this work. Detailed molecular compositions of the sampled particles were determined using a laser desorption ionization (LDI) source coupled with Fourier-transform ion cyclotron resonance mass spectrometry (FT-ICR MS)^38,39^, which has superior mass resolution and accuracy for the molecular characterization of complex mixtures^40,41^. This experimental study presents direct evidence to support the hypothesis that individual PAH molecules grow chemically until they are large enough to agglomerate into orderly stacks and, subsequently, into disordered clusters^33^.

In this work, three different fuels and flame configurations were employed to demonstrate that the experimental evidence is a common phenomenon in the low-temperature nucleation zone, independent of the gas-phase conditions. And the inception progress has a selectivity towards certain PAHs and these PAHs are common across various flames and fuels.

## Results and discussion

**Fig. 1:**
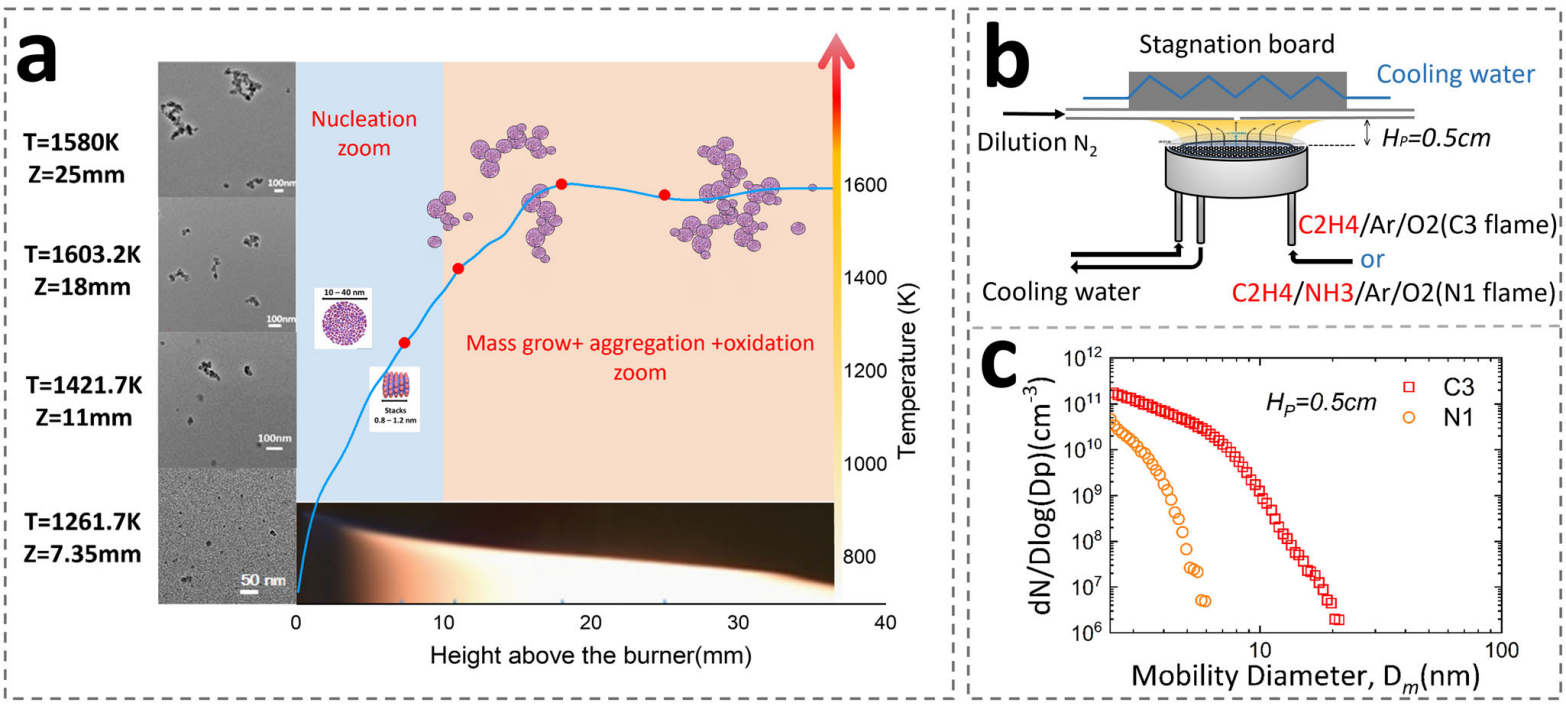
Information about nano-organic carbon particles includes the flames that produced them, sampling locations, and the morphology. **a** The particles are sampled from a nonpremixed flame, the blue line is the temperature profile and the red dots are the sampling positions. TEM images are present for the samples from each sampling point. The nano-organic carbon particles are from the position *z* = 7.35 mm based on their morphology and flame structure. **b** The schematics of the premixed flame and the sampling method. **c** The particle size distribution of the nano-organic carbon particles from premixed flames^42^.

Nonpremixed and premixed flames and three different fuels were considered in this work. Figure 1 presents the three target flames and the soot particle morphology. Figure 1a displays an ethylene-toluene co-flow nonpremixed flame at atmospheric pressure. The NOC particles were sampled using the thermophoresis method at the height above the burner (HAB) of 7.35 mm, which corresponds to an inception region at a temperature of 1261.7 K. According to the transmission electron microscope measurements, all particles were less than 10 nm in size. Figure 1b displays the premixed stagnation flame from which the NOC particles were extracted using a nanometer aerosol sampling instrument coupled with a neutralizer in ethylene and ethylene-ammonia laminar premixed flames at an HAB of 5 mm^42^. The sizes of the NOC particles were measured using a scanning mobility particle sizer (SMPS) system. According to the SMPS results, the particle sizes were 2.4 to 22 nm and 2.4 to 6 nm in ethylene and ethylene-ammonia flame, respectively. Infant soot particles sampled from different flames were composed of nearly the same chemical species, and their probable and stable structures were predicted using a stochastic simulation (SNapS2) and theoretical calculations. The details of the flame parameters, sampling method, and experimental configuration are provided in the Experimental Section. The temperature profiles of the laminar premixed flames are available in Supplementary Fig. S1.2 of Supplementary Note 1.

**Fig. 2:**
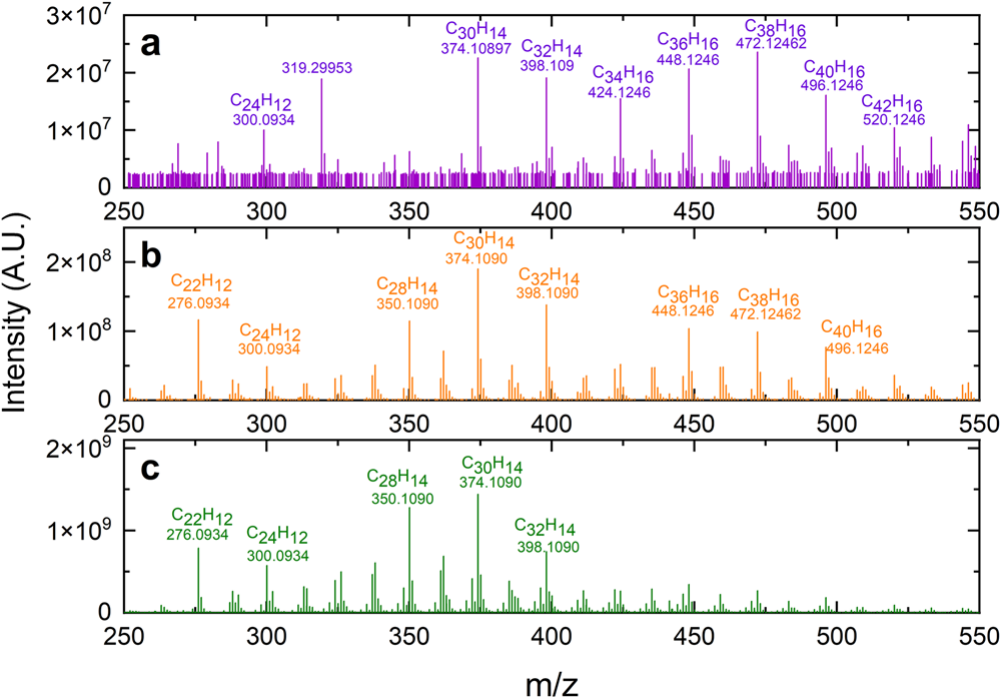
Magnified mass spectra of nano-organic carbon particles. The samples are from a height above the burner of: **a** 7.35 mm in the ethylene-toluene co-flow flame, **b** 5.0 mm in the ethylene laminar premixed flame, **c** 5.0 mm in the ethylene-ammonia laminar premixed flame.

After the NOC particles were found, they were immediately analyzed using LDI coupled with FT-ICR MS. The results in Fig. 2 reveal that 10 predominant PAHs were detected in NOC particles for various flames. In Fig. 2a, eight predominant PAHs were identified in the NOC particles generated in the co-flow flame in the mass range of 300.0924–520.1246 Da. The PAHs captured in the NOC particles from ethylene laminar premixed flames were shifted to a smaller size and fell within the mass range of 276.0934–496.1246 Da (Fig. 2b). For NOC particles in the ethylene-ammonia flame (Fig. 2c), the size of both particles and PAHs (276.0934–398.1090 Da) were smaller than the other two flames. The absolute intensity of the signal is proportional to the quantity of the soot particle sampled, which is related to the local soot density, sampling method, and sampling time. However, due to the various sampling times and methods, the intensity of the PAH signal cannot reflect the actual local soot density across the three targeted flames.

Based on the accurate masses measured by the FT-ICR MS, the chemical formulas of the dominant peaks were identified, and the results are also presented in Fig. 2. Overall, the main compositions of NOC particles among the different flames were similar, and the carbon numbers of the PAHs captured in NOC particles spanned from 22 to 42. To the authors’ knowledge, the PAHs were the smallest among those detected directly from soot particles^20,32–34^, indicating that the NOC particles sampled in this work are from the very beginning of the inception process. In the analysis, all detected PAHs belong to the most stable classes of C_2n_H_12_, C_2n_H_14_, and C_2n_H_16_, as illustrated in Fig. 1 of ref. ^43^. and had the same chemical formulas with molecules referred to as Stein-Fahr’s stabilomers. These stabilomers are a series of PAHs that are the most stable molecules. Their nature and chemical thermodynamic properties were determined using the group additivity estimation method. The most stable classes of C_2n_H_12_, C_2n_H_14_, and C_2n_H_16_ were stable up to 2000 K or even 2500 K, as suggested in ref. ^43^, implying that they survive at the temperatures in the targeted flames. Moreover, the C_30_H_14_ peak exhibited the highest intensity among all NOC particles sampled in the studied flames, indicating that this molecule had the largest proportion of particles.

All identified molecules were PAHs that contained an even number of carbon atoms and hydrogen atoms; the chemical format can be summarized as C_2n_H_2m_. Molecules containing odd numbers of C atoms cannot be completely conjugated and possess relatively high H/C ratios. Thus, they are generally less stable than molecules of a comparable structure containing even numbers of carbon atoms^43^. Furthermore, species with an odd number of H atoms are usually free radicals. In reality, free radicals and species with odd carbon numbers exist in the gas phase of these flames, but the results suggest they are not involved in the inception process. The radicals and species with odd carbon numbers were detected from non-NOC particles (matured particles) sampled at higher flame heights. The detailed mass spectra information of matured particles from premixed flames can be found in the other work^42^, and are available in Supplementary Fig. S2.1 of Supplementary Note 2 for nonpremixed diffusion flames.

**Fig. 3:**
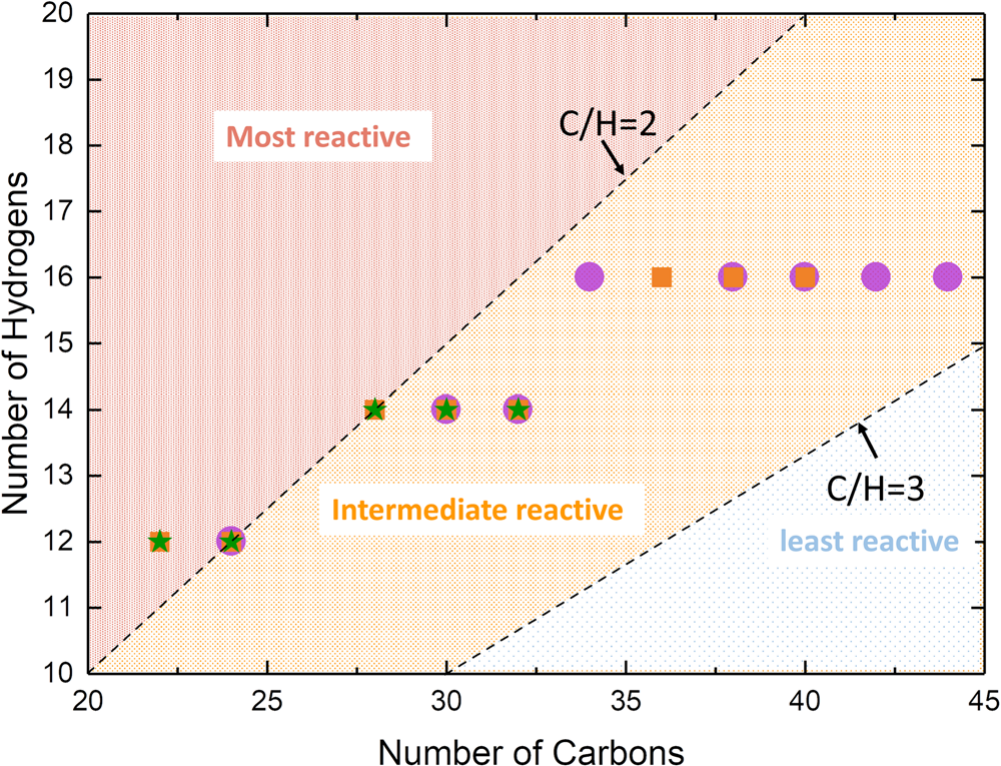
Distribution of the polycyclic aromatic hydrocarbons in nano-organic particles from three targeted flames. Solid purple circles mark the ethylene-toluene co-flow flame in Fig. 2a. Solid orange squares denote the ethylene laminar premixed flame in Fig. 2b. Solid green stars represent the ethylene-ammonia laminar premixed flame in Fig. 2c. The C/H correlation of 2 and 3 distinguishes the PAH reactivity.

Another important observation was that all molecular compositions in NOC particles were hydrocarbons, despite PAHs containing oxygen and nitrogen in the flame reaction region. On the other hand, oxygen and nitrogen were detected in the mature particles. The compound class information of mature particles from premixed flames and nonpremixed flames are provided in Supplementary Fig. S2.2 and Supplementary Fig. S2.3 of Supplementary Note 2, respectively. By calculation, the ratio of the number of carbon atoms (NC) to the number of hydrogen atoms (NH) of the detected PAHs was mostly between 2 and 3, as depicted in Fig. 3 (except for C22H12), indicating that all these were strictly peri-condensed PAHs, without cata-condensed appendages^44,45^. According to the summary by Dias^44^, PAHs with an N_C_/N_H_ ratio between 2 and 3 had intermediate reactivity. Molecules with an N_C_/N_H_ larger than 3 (or smaller than 2) are relatively less reactive (or more reactive), indicating that the global C/H ratio of the NOC particles is also between 2 and 3. According to theoretical calculations, the C/H ratio would be 1.2 to 2 if the NOC particles form via a chemically linked bond between PAHs^16,17,46,47^. However, the C/H ratio of the NOC particles could reach as high as 10 for inception through fullerene-like structures^6^. The C/H ratio of the NOC particles is greater than 2 only when particles are formed through physical dimerization^6,15,19^. Therefore, based on the C/H ratio of the NOC particles in this study, all infant soot particles were generated through physical nucleation.

The molecular structures of PAHs are necessary to further investigate the inception process, but mass spectrometry can only provide the molecular species of each peak in the spectra. How the molecules are formed in the flames remains unknown; therefore, a stochastic modeling code (SNapS2^48,49^) was employed to study the key molecules responsible for inception in the co-flow nonpremixed flame. As demonstrated by the experimental observations, PAHs detected in the NOC particles generated in the co-flow nonpremixed and stagnation-premixed flames were similar, confirmed by the simulation results.

To compare the similarity of the PAHs in both flames, the molecular structures of C_22_H_12_, C_24_H_12_, C_28_H_14_, C_30_H_14_, C_32_H_14_, C_34_H_16_, C_36_H_16_, C_38_H_16_, and C_40_H_16_ were examined from SNapS2 simulation results. The results revealed considerable overlaps of the molecular structures between the two simulated flames. A few possible PAH structures predicted by SNapS2 are provided in Supplementary Table S3.1 of Supplementary Note 3. Five-membered rings were very common for these molecules, in good agreement with the recently revealed molecular structures from atomic force microscopy^50,51^. Normally, PAHs containing five-member rings have a curved structure. In this work, however, almost all SNapS2-generated structures with five-member rings were planar, and no aliphatic chains were found at the edges of the predicted structures. The SNapS2 code obtained molecular structures in the gas phase for the flames, whereas the PAH molecules from the experiment came directly from the soot particles. Therefore a part of SNapS2-generated species could form in the gas phase, but are not major participants in the soot formation process, such as oxygenated PAHs in this study. By investigating the predicted molecules of the peaks confirmed by experiments, SNapS2 provides possible molecular structures and their reaction pathways, which help reveal the inception process.

In addition to the molecular structures predicted by SNapS2, the structures with the highest thermodynamic stability were determined using quantum chemical calculation and group additivity methods using the *reaction mechanism generator*^52^ were also presented in Supplementary Note 3. The Gibbs free energy of isomers for each detected PAH was calculated at a temperature of 1261.7 K, and the structure with the lowest Gibbs free energy was considered that with the highest thermodynamic stability. Because it was the local temperature of the NOC particles in the co-flow nonpremixed flames, 1261.7 K was chosen. However, soot particles from the laminar premixed flame were sampled from the probe embedded in the stagnation plate. A sharp temperature decline occurred when the plate was approached, making it difficult to identify the local temperature of the NOC particles generated in this type of stagnation-premixed flame. Due to the high cost and computational complexity, the thermodynamic properties of larger molecules were calculated using the reaction mechanism generator. The detailed methodologies of this quantum chemical calculation and group additivity method are available in Supplementary Note 4 and the most thermodynamic stability structure are available in Supplementary Note 3. In general, PAH isomers containing five-member rings usually have higher Gibbs free energy by calculation, and peri-condensed PAHs with only six-member rings are the most stable. According to the theoretical calculations, the most thermodynamically stable molecular structures are consistent with stabilomers. For comparison, Supplementary Note 3 lists the most probable and thermodynamically stable structures of PAHs detected in the NOC particles using SNapN2 prediction, the current theoretical calculation, and those from Stein-Fahr^43^.

Based on the experimental observation and theoretical calculation, the characteristics of the PAHs in NOC particles are summarized as follows. First, the molecular weight is between 270 and 520 Da, which is the medium size among the known PAHs contributing to soot formation^6,53^. Second, these PAHs have planar structures with no aliphatic chain additive. Last, these molecules are pure hydrocarbons; no molecules containing oxygen or nitrogen were observed. These specific PAHs with these structures could easily agglomerate into stacked clusters and then disordered clusters^14,23,33^, providing robust evidence supporting the physical nucleation hypothesis. These key findings are assessed in light of the existing studies.

### PAH size

Frenklach and Wang^11^ proposed a hypothesis 30 years ago that the dimerization of pyrene would be the initial step for soot inception. Following that hypothesis, many soot models^54–56^ have adopted the physical dimerization (involving van der Waals forces) of pyrene as the beginning of inception. However, recent experimental and kinetic evidence has indicated that pyrene dimers are unlikely to play a key role in the soot inception process due to their disassociation and short lifetime in combustion environments^22,57^. Because of stronger physical interactions^14,21,58–60^, large PAH monomers are more likely to grow into incipient soot particles than small PAHs. In this experiment, the smallest PAH detected in the NOC particle was C_24_H_12_ (A7, which contains seven benzene rings) from the co-flow flame and C_22_H_12_ (A6) from the laminar premixed flame. Both had much higher dimerization binding energy and equilibrium than pyrene.

In this study, PAH captured with the largest molecular weight was C_42_H_16_ (A14). Molecules of a larger size were rarely generated in the relatively low-temperature inception region. Based on these results, a greater possibility exists of moderate-sized PAHs forming dimers in the inception region and surviving. According to ref. ^58^, the size of the molecule is the second parameter affecting dimerization after temperature.

### PAH structure

In addition to the size of the PAH monomer, the structures of PAHs (sometimes called polycyclic aromatic compounds or PACs), including the presence of oxygen and aliphatic side chains and shapes also play a significant role in affecting physical nucleation efficiency. In the environment of the targeted flame, the oxygen-contained PACs and the nitrogen-contained PACs can be generated in the gas phase. However, only pure hydrocarbons were observed in the NOC particles in this experiment, possibly because PAH dimers containing oxygen have greater free energy than those without oxygen for a similar monomer size^58^. A similar explanation applies to PAHs containing nitrogen, but no research exists regarding their physical dimerization, and additional work is needed to confirm this. Thus, pure hydrocarbon PAHs become dimers more easily than those containing oxygen or nitrogen. Previous experimental studies^20,50,51,53^ have provided evidence of an aliphatic side chain in nascent soot particles. However, molecular dynamic simulation suggests that the presence of an aliphatic side chain did not facilitate dimerization^58^, which is consistent with the predicted structures in this work. Regarding the shape, most PAH structures predicted by SNapN2 contained five-member rings but were planar, not curved. The curved structure of PAHs negatively affects dimerization, but it is still less important than the presence of oxygen and side chains^58,61^. Overall, the structure of PAHs predicted in this work favors physical dimerization.

### Temperature limitation

Temperature is one of the most important parameters affecting soot inception and growth. In the ethylene-toluene co-flow nonpremixed flame, the local temperature of the sampled NOC particles was 1261.7 K (988.55 °C). For the investigated laminar premixed flames, the maximum temperature was 1858 K. As mentioned, the local temperature of the NOC particles sampled in those two flames could not be identified. According to the inception mechanisms in Fig. 10 in ref. ^21^., the detected PAHs in this work could only form NOC particles through physical nucleation at the targeted flame temperature. Chemical inception only occurs if the temperature surpasses 2500 K^21^. The local temperature of the sampled NOC particles in the co-flow flame is close to the temperature of PAH thermal isomerization conditions^62^, suggesting that the PAHs predicted by SNapN2 in the gas phase were most likely transformed to stabilomers via thermal isomerization.

**Fig. 4:**
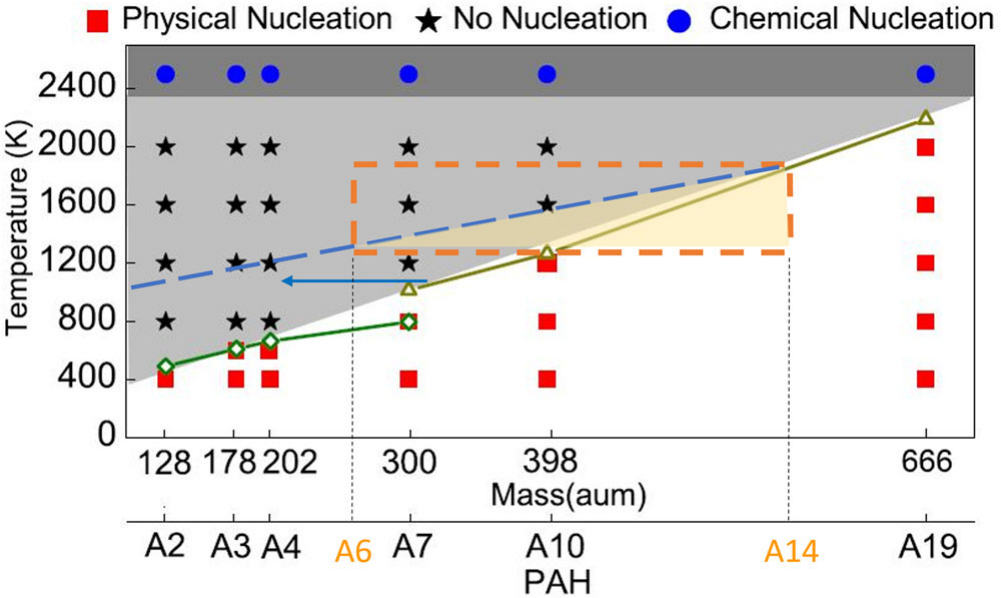
Inception mechanisms of polycyclic aromatic hydrocarbons in homogenous systems^21^. The dashed line parallels the physical nucleation limitation line, estimated considering heterodimerization. The orange box represents the temperature and PAH size limitations. The yellow area is the physical nucleation possibility. A6 indicates polycyclic aromatic hydrocarbon contains six benzene rings.

Of all parameters, the PAH size and environmental temperature are the most important parameters for inception^6,58^. Based on the inception mechanism provided in ref. ^21^, in this case, the inception region was outlined according to the size of the detected PAHs and flame temperatures, as illustrated in Fig. 4. The original physical nucleation limit was calculated based on homogenous systems, implying that only the self-dimerization of individual PAH molecules was considered.

When a system contains molecules with a wide variety of sizes and shapes, collision and dimerization between different molecules occurs, which is called heterodimerization^63^. According to the van der Waals force, the self-dimer formation of Molecule A is smaller than in the dimer of Molecule A with Molecule B if Molecule B is larger than Molecule A, which indicates that the dimer of Molecule A with Molecule B survives more easily than the self-dimer of Molecule A in the same conditions. The physical nucleation zone was widened by shifting the physical nucleation line toward the estimated small size, considering the heterodimerization^63^. The region of physical nucleation observed in this study can be bound by temperature, PAH size (orange rectangle), and the heterodimerization limit, resulting in the yellow-shaded subregion in Fig. 4.

### Conclusions

This work employed state-of-the-art experimental and modeling techniques to provide direct evidence on solving the selectivity of the soot inception process. Using ultrahigh-resolution FT-ICR MS with a LDI source, PAH structural information of very early-stage NOC particles was obtained in different flames. The secondary collisions of the sampled particles and PAHs were minimized using this method, enabling the direct study of the selected particles participating in the inception process. Similar PAHs were identified using experiments and SNapS2 simulations regardless of flame type. The molecular weight of these PAHs spanned from 276 to 520 Da. Condensed PAHs with even-numbered carbon and hydrogen atoms played a key role. The SNapS2 simulation and theoretical calculations provided the most plausible and stable structures for each PAH peak. The selected structures, within a moderate size at the given flame temperature region, represent the most favorable species involved in physical dimerization, providing direct and solid evidence that soot inception predominantly begins with a physical process.

## Methods

### Co-flow nonpremixed flame and soot sampling

The atmospheric-pressure laminar co-flow nonpremixed burner assembly details are briefly described. Experiments were performed on a Santoro^64^ burner, consisting of two concentric brass tubes of 11.1 and 101.6 mm i.d., with fuel flowing through the central tube and a co-flow of air in the outer tube. Liquid fuel was supplied from a syringe pump to a mixture chamber, where it was vaporized and mixed with a carrier gas (ethylene and nitrogen) at 423 K. Carrier gases were controlled using mass flow controllers. The flow rates of the liquid fuel and carrier gases were 0.845 mL/h and 120 SCCM, respectively. Air co-flow was also controlled using an MKS mass flow controller with a flow rate of 10 SLPM.

Soot was sampled from the flame using thermophoretic sampling on pure copper grids (Electron Microscopy Science, GA75-Cu) at four positions, as depicted in Fig. 1. The sampling locations of the four positions were at 7.35, 11, 18, and 25 mm in height above the burner (HAB). The sampling system details are described in ref. ^65^. The sampling device was designed to insert the probe through the flame. The flame was extinguished immediately afterward, and the grid was retrieved. A high-speed camera measured the residence time of the grid in the flame. The details of the nonpremixed flame configuration and the sampling process were provided in Supplementary Fig. S1.1 of Supplementary Note 1. The exposure time was limited to ~7 µs for all sampling positions to minimize the coagulation effect during the sampling process^66^. The temperature profile of the central line was measured using a type-B thermocouple with a radiation correction.

### Laminar premixed flame and soot sampling

The laminar premixed flame configuration at KAUST consists of a McKenna burner with a diameter of 60 mm and a stagnation board parallel to the burner surface, also called a burner stagnation stabilization flame. The setup is similar to that used in other work^37,67,68^. The details of this setup have been described in ref. ^42^. The flow rates of the unburned gas were controlled with MKS mass flow controllers. Generated soot particles in the flame were inhaled into a sampling orifice with a diameter of 138 µm, and the orifice was in the center of a sampling tube embedded in the stagnation plate. The temperature of the orifice (about 400 K) was measured using a K-type thermocouple embedded within the stagnation plate. Once the particles entered the orifice, they were immediately diluted by a high flow of cold nitrogen (30 L/min, STP) to minimize losses in the sampling lines due to coagulation. The dilution ratio in this work was calibrated by measuring the flow rate into the orifice from the ambient air, as described in ref. ^37^. The optimal dilution ratio was determined using the procedure described in ref. ^37^. by adjusting the secondary air flow rate.

After dilution, some soot particles were analyzed with the scanning mobility particle sizer (SMPS, TSI 3936) to measure the particle size distribution. The SMPS system consisted of a neutralizer (TSI 3087), nano-differential mobility analyzer (DMA, TSI 3086), and ultrafine condensation particle counter (CPC, TSI 3776). Because particle sizes smaller than 10 nm are overestimated (due to the Cunningham slip correction, as discussed by Li et al.^69^), the diameter of the soot particles was corrected based on the particle transport theory presented in ref. ^70^.

In addition, diluted soot particles were charged by the neutralizer (TSI 3087) and collected using a nanometer aerosol sampling instrument (NAS, TSI 3089). The NAS consisted of a cylindrical sampling chamber and electrode with a flat round plate perpendicularly mounted to the aerosol flow^71^. In this work, the flow rate through the NAS and the voltage were controlled at 1.5 L/min and –7 kV, respectively. Positively charged soot particles were captured on the substrate of negatively charged pure copper grids (Electron Microscopy Science, GA75-Cu).

### Soot samples analyzed using Fourier-transform ion cyclotron resonance mass spectrometry

The molecular characterization of the collected NOC particles on copper grids was performed on a 9.4 Tesla SolariX XR Fourier-transform ion cyclotron resonance mass spectrometer (FT-ICR MS; Bruker Daltonik GmbH, Bremen, Germany) equipped with laser desorption ionization (LDI) at the Analytical Chemistry Core Laboratory in KAUST. It was applied to examine the chemical compositions of the collected soot particles. The mass spectrometer was previously calibrated with the electrospray ionization source using a standard solution sodium format in the mass range of 100–1200 Da. The instrument was operated under the data size of 4 M, with a transient time of 2.2370 s.

For the FT-ICR MS measurements, copper support grids containing soot samples were attached to the LDI sample plate with double-sided adhesive tape. The LDI with a laser type: BRUKER *smartbeam*^TM^ -II laser, 355 nm wavelength (3.5 billion laser shots, 2 kHz repetition rate, ≥10 µJ/pulse laser energy). For LDI, the pulse energy was less than 500 µJ, and the diameter of the beam size was 1.5 mm. The maximum laser energy was 28.3 mJ cm^–2^. The laser energy was tuned for each sample before acquiring the final spectrum. Minimal laser energy was employed to avoid an apparent fragmentation of molecular ions and the formation of carbon clusters. One hundred individual mass scans were accumulated into one final spectrum to generate profound mass signals and produce results more representative of the entire specimen by sampling different locations on the copper grid. The lowest applied laser energy in this experiment was around 20% of the maximum energy (~5.66 mJ cm^–2^).

The resulting mass spectra were further processed using DataAnalysis software (v. 4.5). Only the mass peaks with a high signal-to-noise ratio (SNR ≥ 5) were considered for the following molecular formula identification. Composer software (Sierra Analytics, Modesto, CA, USA) was employed to assign a unique chemical formula to a typical soot sample (C_1-100_H_2-200_N_0-4_O_0-2_).

### PAH structure prediction using SNapS2 and CFD

A stochastic modeling code (SNapS2) was employed to simulate the formation process of the PAHs in this co-flow nonpremixed flame to further examine the gas-phase molecules responsible for the inception process. As input, SNapS2 takes gas-phase environments (small-species mole fractions and temperature profiles) and provides the time evolution (traces) of the seed molecules along the flame. The SNapS2 code uses the kinetic Monte Carlo scheme and generic reactions described by the atomic chemical and physical neighborhoods (reactive sites). The advantage of stochastic modeling is that the molecules continue to evolve along the flame. Unlike deterministic modeling, in which species are predefined, the SNapS2 code enables the molecules to grow into new species that can be used to explore molecular structures. Compared with its previous version SNAPS, the SNapS2 code is more efficient to enable complex 2D flame simulations, and its mechanism is more comprehensive. The 430 generic reactions in the SNapS2 kinetic mechanism were fully reversible, thermodynamically consistent, and sterically resolved, including major PAH growth pathways. Over the past few years, the SNapS2 code and its mechanism have been validated against experimental measurements in the mass spectrum, oxygen content, and molecular structures, and it has proven its ability to produce valuable information on different combustion systems.

For this co-flow nonpremixed flame, the temperature profile and mole fraction profiles for species with a molar mass less than benzene were measured and provided as input parameters for a computational fluid dynamics (CFD) simulation. This simulation was performed using a solver employing a low-Mach algorithm with modifications to ensure mass conservation for combustion-related problems. The computational domain was subdivided into 91 cells in the radial direction and 380 in the axial direction to form a structured, nonuniform mesh with 33,960 cells. The simulated temperature profile and main products along the central line using the CFD calculation agree well with the experimental data. The details of the CFD simulation of the co-flow nonpremixed flame are supplied in Supplementary Note 5. The centerline of the CFD results was used as input for the SNapS2 simulations. Ten thousand molecules were simulated using benzene as the seed molecule. The pure hydrocarbon mechanism, which excluded oxygen chemistry, was used to improve the computational performance for the SNapS2 simulations because the oxygenated PAHs were less than 5% from the compound class distribution analysis at an HAB of 7.35 mm in this flame.

The observations of the experiment also demonstrated that the PAHs formed at an HAB of 5 mm in a laminar premixed flame were very similar to those formed at an HAB of 7.35 mm in this co-flow nonpremixed flame. The simulations were conducted to confirm this finding. The system was atmospheric-pressure fuel-rich ethylene laminar premixed flame with a mole fraction of ethylene/oxygen/argon = 0.163/0.237/0.6. The gas-phase environment of this flame was modeled with a PREMIX code in CHEMKIN^72^ software with the KM2 mechnisam^73^. The temperature profile of this laminar premixed flame was experimentally measured and input into the CHEMKIN simulation to improve the heat modeling accuracy. The gas-phase environment was input into the SNapS2 code to study PAH formation in this laminar premixed flame. Finally, 10,000 molecules were simulated using the pure hydrocarbon mechanism and benzene as the seed molecule (for the previously discussed reason).

## Supplementary information

Supplementary Information

## Data Availability

Extra experimental details can be found in the Supplementary Information. The data that support the findings of this study are available from the corresponding author upon reasonable request.

## References

[1] Karagulian, F. et al. Contributions to cities’ ambient particulate matter (PM): a systematic review of local source contributions at global level. *Atmos. Environ.* **120**, 475–483 (2015).

[2] Pant, P. & Harrison, R. M. Estimation of the contribution of road traffic emissions to particulate matter concentrations from field measurements: a review. *Atmos. Environ.* **77**, 78–97 (2013).

[3] Lohmann, U. et al. Future warming exacerbated by aged-soot effect on cloud formation. *Nat. Geosci.* **13**, 674–680 (2020).

[4] Antiñolo, M., Willis, M. D., Zhou, S. & Abbatt, J. P. Connecting the oxidation of soot to its redox cycling abilities. *Nat. Commun.* **6**, 1–7 (2015). 10.1038/ncomms7812 PMC4410628 25873384

[5] Park, S.-R. et al. The impact of fine particulate matter (PM) on various beneficial functions of human endometrial stem cells through its key regulator SERPINB2. *Exp. Mol. Med*. **53**, 1850–1865 (2021). 10.1038/s12276-021-00713-9 PMC8741906 34857902

[6] Wang, H. Formation of nascent soot and other condensed-phase materials in flames. *Proc. Combust. Inst.* **33**, 41–67 (2011).

[7] Gatchell, M. et al. Survival of polycyclic aromatic hydrocarbon knockout fragments in the interstellar medium. *Nat. Commun.* **12**, 1–8 (2021). 34789760 10.1038/s41467-021-26899-0 PMC8599666

[8] Knacke, R. Carbonaceous compounds in interstellar dust. *Nature* **269**, 132–134 (1977).

[9] D’Anna, A. & Violi, A. A kinetic model for the formation of aromatic hydrocarbons in premixed laminar flames. in *Symposium (International) on Combustion*. 425-433 (Elsevier, 1998).

[10] Wang, H. & Frenklach, M. A detailed kinetic modeling study of aromatics formation in laminar premixed acetylene and ethylene flames. *Combust. Flame* **110**, 173–221 (1997).

[11] Frenklach, M. & Wang, H. Detailed modeling of soot particle nucleation and growth. In *Symposium (International) on Combustion*. 1559–1566 (Elsevier, 1991).

[12] Frenklach, M. New form for reduced modeling of soot oxidation: accounting for multi-site kinetics and surface reactivity. *Combust. Flame* **201**, 148–159 (2019).

[13] Homann, K. H. Fullerenes and soot formation—new pathways to large particles in flames. *Angew. Chem. Int. Ed.* **37**, 2434–2451 (1998). 10.1002/(SICI)1521-3773(19981002)37:18<2434::AID-ANIE2434>3.0.CO;2-L 29711358

[14] Herdman, J. D. & Miller, J. H. Intermolecular potential calculations for polynuclear aromatic hydrocarbon clusters. *J. Phys. Chem. A* **112**, 6249–6256 (2008). 18572902 10.1021/jp800483h

[15] Frenklach, M. & Wang, H. Detailed modeling of soot particle nucleation and growth. *Proc. Combust. Inst.* **23**, 1559–1566 (1991).

[16] Violi, A. Modeling of soot particle inception in aromatic and aliphatic premixed flames. *Combust. Flame* **139**, 279–287 (2004).

[17] Chung, S. & Violi, A. Insights on the formation and growth of carbonaceous nanoparticles in high temperature environments. In *Combustion generated fine carbonaceous particles*. (eds Bockhorn, H., D'Anna, A., Sarofim, A. F. & Wang, H.) pp 321–332 (KIT Scientific Publishing, 2009).

[18] *Combustion Generated Fine Carbonaceous Particles*: *Proceedings of an International Workshop Held in Villa Orlandi*, *Anacapri, May 13–16, 2007* (KIT Scientific Publishing, 2009).

[19] Frenklach, M. On the driving force of PAH production. in *Symposium (International) on Combustion* 1075–1082 (Elsevier, 1989).

[20] Cain, J., Laskin, A., Kholghy, M. R., Thomson, M. J. & Wang, H. Molecular characterization of organic content of soot along the centerline of a coflow diffusion flame. *Phys. Chem. Chem. Phys.* **16**, 25862–25875 (2014). 25354231 10.1039/c4cp03330b

[21] Mao, Q., van Duin, A. C. & Luo, K. Formation of incipient soot particles from polycyclic aromatic hydrocarbons: a ReaxFF molecular dynamics study. *Carbon* **121**, 380–388 (2017). 10.1039/c9cp00354a 31033994

[22] Sabbah, H., Biennier, L., Klippenstein, S. J., Sims, I. R. & Rowe, B. R. Exploring the role of PAHs in the formation of soot: pyrene dimerization. *J. Phys. Chem. Lett.* **1**, 2962–2967 (2010).

[23] Miller, J. H. The kinetics of polynuclear aromatic hydrocarbon agglomeration in flames. in *Symposium (International) on Combustion*. 91–98 (Elsevier, 1991).

[24] D’Anna, A., D’Alessio, A. & Minutolo, P. Spectroscopic and chemical characterization of soot inception processes in premixed laminar flames at atmospheric pressure. In *Soot formation in combustion: mechanisms and models*. (ed Bockhorn, H.) pp 83–103 (Springer 1994).

[25] Dobbins, R. A. & Subramaniasivam, H. Soot precursor particles in flames. In *Soot formation in combustion: mechanisms and models*. (ed Bockhorn, H.) pp 290–301 (Springer, 1994).

[26] D’Anna, A., Rolando, A., Allouis, C., Minutolo, P. & D’Alessio, A. Nano-organic carbon and soot particle measurements in a laminar ethylene diffusion flame. *Proc. Combust. Inst.* **30**, 1449–1456 (2005).

[27] D’Anna, A., Commodo, M., Violi, S., Allouis, C. & Kent, J. Nano organic carbon and soot in turbulent non-premixed ethylene flames. *Proc. Combust. Inst.* **31**, 621–629 (2007).

[28] Kholghy, M., Saffaripour, M., Yip, C. & Thomson, M. J. The evolution of soot morphology in a laminar coflow diffusion flame of a surrogate for Jet A-1. *Combust. Flame* **160**, 2119–2130 (2013).

[29] Dobbins, R., Fletcher, R. & Lu, W. Laser microprobe analysis of soot precursor particles and carbonaceous soot. *Combust. Flame* **100**, 301–309 (1995).

[30] Dobbins, R., Fletcher, R. A. & Chang, H.-C. The evolution of soot precursor particles in a diffusion flame. *Combust. Flame* **115**, 285–298 (1998).

[31] Fletcher, R. A., Dobbins, R. & Chang, H.-C. Mass spectrometry of particles formed in a deuterated ethene diffusion flame. *Anal. Chem.* **70**, 2745–2749 (1998). 21644789 10.1021/ac971293q

[32] Sabbah, H. et al. Molecular content of nascent soot: family characterization using two-step laser desorption laser ionization mass spectrometry. *Proc. Combust. Inst.* **38**, 1241–1248 (2021). 10.1016/j.proci.2020.09.022 PMC7610591 33850480

[33] Jacobson, R. S., Korte, A. R., Vertes, A. & Miller, J. H. The molecular composition of soot. *Angew. Chem.* **132**, 4514–4520 (2020). 10.1002/anie.201914115 31917890

[34] Faccinetto, A. et al. Evidence on the formation of dimers of polycyclic aromatic hydrocarbons in a laminar diffusion flame. *Commun. Chem.* **3**, 1–8 (2020). 36703341 10.1038/s42004-020-00357-2 PMC9814144

[35] Adamson, B. A., Skeen, S. A., Ahmed, M. & Hansen, N. Nucleation of soot: experimental assessment of the role of polycyclic aromatic hydrocarbon (PAH) dimers. *Z. Phys. Chem.* **234**, 1295–1310 (2020).

[36] Adamson, B., Skeen, S., Ahmed, M. & Hansen, N. Detection of aliphatically bridged multi-core polycyclic aromatic hydrocarbons in sooting flames with atmospheric-sampling high-resolution tandem mass spectrometry. *J. Phys. Chem. A* **122**, 9338–9349 (2018). 30415549 10.1021/acs.jpca.8b08947

[37] Camacho, J. et al. Mobility size and mass of nascent soot particles in a benchmark premixed ethylene flame. *Combust. Flame* **162**, 3810–3822 (2015).

[38] Zhang, W. & Müllen, K. Analyzing solid fossil-fuel pitches by a combination of Soxhlet extraction and Fourier transform ion cyclotron resonance mass spectrometry. *Carbon* **167**, 414–421 (2020).

[39] Zhang, W., Shao, C. & Sarathy, S. M. Analyzing the solid soot particulates formed in a fuel-rich flame by solvent-free matrix-assisted laser desorption/ionization Fourier transform ion cyclotron resonance mass spectrometry. *Rapid Commun. Mass Spectrom.* **34**, e8596 (2020). 31756786 10.1002/rcm.8596

[40] Smith, D. F., Podgorski, D. C., Rodgers, R. P., Blakney, G. T. & Hendrickson, C. L. 21 tesla FT-ICR mass spectrometer for ultrahigh-resolution analysis of complex organic mixtures. *Anal. Chem.* **90**, 2041–2047 (2018). 29303558 10.1021/acs.analchem.7b04159

[41] Marshall, A. G. & Rodgers, R. P. Petroleomics: the next grand challenge for chemical analysis. *Acc. Chem. Res.* **37**, 53–59 (2004). 14730994 10.1021/ar020177t

[42] Shao, C. et al. Effects of ammonia addition on soot formation in ethylene laminar premixed flames. *Combust. Flame* **235**, 111698 (2022).

[43] Stein, S. E. & Fahr, A. High-temperature stabilities of hydrocarbons. *Am. J. Phys. Chem.* **89**, 3714–3725 (1985).

[44] Dias, J. R. Isomer enumeration of nonradical strictly peri-condensed polycyclic aromatic hydrocarbons. *Can. J. Chem.* **62**, 2914–2922 (1984).

[45] Trinajstic, N., Jericevic, Ž., Knop, J., Muller, W. & Szymanski, K. Computer generation of isomeric structures. *Pure Appl. Chem.* **55**, 379–390 (1983).

[46] D’Anna A. Particle inception and growth: experimental evidences and a modeling attempt. In *Combustion generated fine carbonaceous particles* (eds Bockhorn, H., D'Anna, A., Sarofim, A. F. & Wang, H.) pp 299–320 (KIT Scientific Publisher, 2009).

[47] Martin, J. W., Salamanca, M. & Kraft, M. Soot inception: carbonaceous nanoparticle formation in flames. *Prog. Energy Combust. Sci.* **88**, 100956 (2022).

[48] Wang, Q. et al. Spatial dependence of the growth of polycyclic aromatic compounds in an ethylene counterflow flame. *Carbon* **149**, 328–335 (2019).

[49] Wang, Q., Saldinger, J. C., Elvati, P. & Violi, A. Molecular structures in flames: a comparison between SNapS2 and recent AFM results. *Proc. Combust. Inst.* **38**, 1133–1141 (2021).

[50] Schulz, F. et al. Insights into incipient soot formation by atomic force microscopy. *Proc. Combust. Inst.* **37**, 885–892 (2019).

[51] Commodo, M. et al. On the early stages of soot formation: molecular structure elucidation by high-resolution atomic force microscopy. *Combust. Flame* **205**, 154–164 (2019).

[52] Allen, J. W., Goldsmith, C. F. & Green, W. H. Automatic estimation of pressure-dependent rate coefficients. *Phys. Chem. Chem. Phys.* **14**, 1131–1155 (2012). 22146884 10.1039/c1cp22765c

[53] Cain, J. P., Camacho, J., Phares, D. J., Wang, H. & Laskin, A. Evidence of aliphatics in nascent soot particles in premixed ethylene flames. *Proc. Combust. Inst.* **33**, 533–540 (2011).

[54] Kazakov, A., Wang, H. & Frenklach, M. Detailed modeling of soot formation in laminar premixed ethylene flames at a pressure of 10 bar. *Combust. Flame* **100**, 111–120 (1995).

[55] Bai, X., Balthasar, M., Mauss, F. & Fuchs, L. Detailed soot modeling in turbulent jet diffusion flames. *Symp. (Int.) Combust.* **27**, 1623–1630 (1998).

[56] Mosbach, S. et al. Towards a detailed soot model for internal combustion engines. *Combust. Flame* **156**, 1156–1165 (2009).

[57] Chakraborty, D., Lischka, H. & Hase, W. L. Dynamics of pyrene-dimer association and ensuing pyrene-dimer dissociation. *J. Phys. Chem. A* **124**, 8907–8917 (2020). 33064487 10.1021/acs.jpca.0c06677

[58] Elvati, P., Turrentine, K. & Violi, A. The role of molecular properties on the dimerization of aromatic compounds. *Proc. Combust. Inst.* **37**, 1099–1105 (2019).

[59] Lowe, J. S., Lai, J. Y., Elvati, P. & Violi, A. Towards a predictive model for polycyclic aromatic hydrocarbon dimerization propensity. *Proc. Combust. Inst.* **35**, 1827–1832 (2015).

[60] Elvati, P. & Violi, A. Thermodynamics of poly-aromatic hydrocarbon clustering and the effects of substituted aliphatic chains. *Proc. Combust. Inst.* **34**, 1837–1843 (2013).

[61] Chung, S.-H. & Violi, A. Peri-condensed aromatics with aliphatic chains as key intermediates for the nucleation of aromatic hydrocarbons. *Proc. Combust. Inst.* **33**, 693–700 (2011).

[62] Scott, L. T. Exotic chemistry and rational organic syntheses at 1000 °C. *J. Org. Chem.* **81**, 11535–11547 (2016). 27934463 10.1021/acs.joc.6b02113

[63] Eaves, N. A., Dworkin, S. B. & Thomson, M. J. Assessing relative contributions of PAHs to soot mass by reversible heterogeneous nucleation and condensation. *Proc. Combust. Inst.* **36**, 935–945 (2017).

[64] Santoro, R., Semerjian, H. & Dobbins, R. Soot particle measurements in diffusion flames. *Combust. Flame* **51**, 203–218 (1983).

[65] Cenker, E., Bennett, A. & Roberts, W. L. Investigations of the long-term effects of LII on soot and bath gas. *Aerosol Sci. Technol.* **51**, 1354–1367 (2017).

[66] Dobbins, R. & Megaridis, C. Morphology of flame-generated soot as determined by thermophoretic sampling. *Langmuir* **3**, 254–259 (1987).

[67] Lin, B. et al. Effect of mixing methane, ethane, propane and ethylene on the soot particle size distribution in a premixed propene flame. *Combust. Flame* **193**, 54–60 (2018).

[68] Shao, C. et al. Effect of methane doping on nascent soot formation in ethylene-based laminar premixed flames. *Fuel* **186**, 422–429 (2016).

[69] Li, Z., Wang, H., Shandakov, S. D., Nasibulin, A. G. & Kauppinen, E. I. Comment on: Phenomenological description of mobility of nm-and sub-nm-sized charged aerosol particles in electric field by et al., Shandakov, SD, Nasibulin, AG and Kauppinen, EI Author’s reply. *J. Aerosol Sci.* **37**, 111–118. (2006).

[70] Abid, A. D., Camacho, J., Sheen, D. A. & Wang, H. Quantitative measurement of soot particle size distribution in premixed flames-the burner-stabilized stagnation flame approach. *Combust. Flame* **156**, 1862–1870 (2009).

[71] Tang, Q., Ge, B., Ni, Q., Nie, B. & You, X. Soot formation characteristics of n-heptane/toluene mixtures in laminar premixed burner-stabilized stagnation flames. *Combust. Flame* **187**, 239–246 (2018).

[72] ANSYS. CHEMKIN-PRO 2023 R1. 2023.

[73] Wang, Y., Raj, A. & Chung, S. H. A PAH growth mechanism and synergistic effect on PAH formation in counterflow diffusion flames. *Combust. Flame* **160**, 1667–1676 (2013).

