# Supplementary material for: Elucidating the polycyclic aromatic hydrocarbons involved in soot inception
Source: Commun Chem. 2023 Oct 16;6:223. doi: 10.1038/s42004-023-01017-x (PMC10579345; doi:10.1038/s42004-023-01017-x)
Supplement: Supplementary file 1 — Supplementary Information [file 42004_2023_1017_MOESM1_ESM.pdf]

Supplementary Information to

Elucidating the polycyclic aromatic hydrocarbons involved in soot inception

Can Shao[1] † *, Qi Wang[2] †, Wen Zhang[3]*, Anthony Bennett[1], Yang Li[4,1], Junjun Guo[1], Hong G. Im[1], William L. Roberts[1], Angela Violi[2, 5, 6], Subram Mani Sarathy[1]*

[1]King Abdullah University of Science and Technology (KAUST), Clean Combustion Research Center, Thuwal, 23955-6900, Saudi Arabia

[2]University of Michigan, Department of Mechanical Engineering, 2350 Hayward St, Ann Arbor, MI 48109-2125, United States

[3]King Abdullah University of Science and Technology (KAUST), Core Labs, Thuwal, 23955-6900, Saudi Arabia

[4]Science and Technology on Combustion, Internal Flow and Thermostructure Laboratory, School of Astronautics, Northwestern Polytechnical University, Xi'an 710072, China

[5]University of Michigan, Department of Chemical Engineering, 2350 Hayward St, Ann Arbor, MI 48109-2125, United States

[6]University of Michigan, Chemical Engineering and Biophysics Program, 930 N. University Ave, Ann Arbor, MI 48109-1055, United States

Correspondence:

Can Shao, King Abdullah University of Science and Technology (KAUST), Clean Combustion Research Center, Thuwal, 23955-6900, Saudi Arabia. Email: can.shao@kaust.edu.sa

Wen Zhang, King Abdullah University of Science and Technology (KAUST), Core Labs, Thuwal, 23955-6900, Saudi Arabia. Email: zhangw.chem@gmail.com

S. Mani Sarathy, King Abdullah University of Science and Technology (KAUST), Clean Combustion Research Center, Thuwal, 23955-6900, Saudi Arabia. Email: mani.sarathy @kaust.edu.sa

†These authors contributed equally.

Contents

Supplementary Note 1: Additional details of the target flames **3**
Supplementary Note 2: Additional MS information of matured soot particles. **5**
Supplementary Note 3: The structures of PAHs predicted by various methods. **8**
Supplementary Note 4: Theoretical Prediction of Thermochemistry **10**
4.1 Quantum Chemical Calculation 10
4.2 Group Additivity Method Calculation 10
Supplementary Note 5: The CFD simulation of nonpremixed flame. **11**
5.1 Numerical model and computational configuration 11
5.2 Simulation results 12
Supplementary references **14**

Supplementary Note 1: Additional details of the target flames.

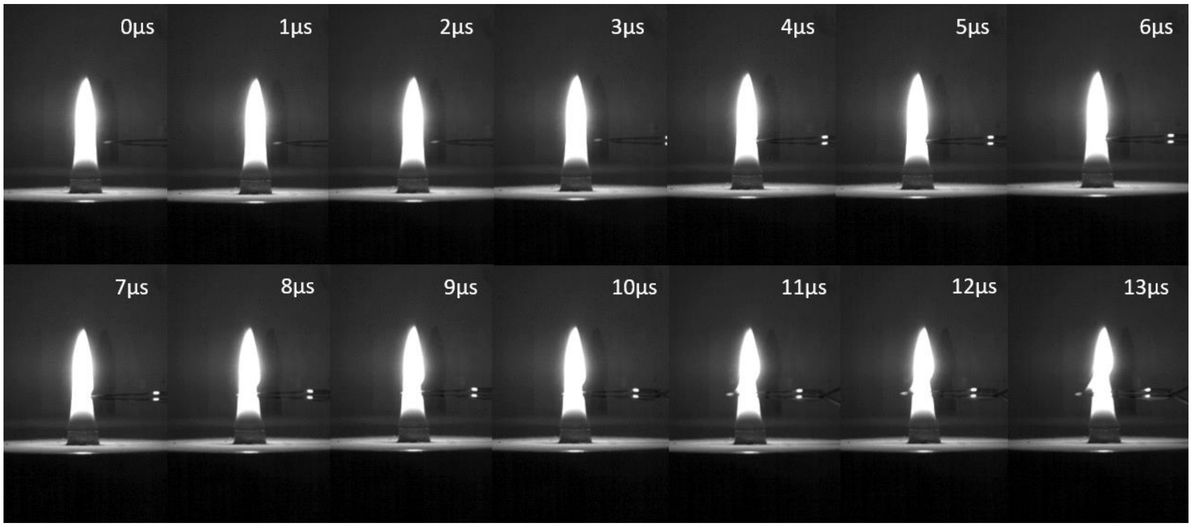

Figure S1.1: Nonpremixed flame configuration and sampling process.

Table S1.1. Summary of premixed flames conditions[a].

Flame	Mole fraction				Equivalence ratio	T_{max}(K)[b]
	C_2H_4	O_2	Ar	NH_3		
C3[1,2]	0.163	0.237	0.6	0	2.063	1859±75K
N1[3]	0.163	0.237	0.5837	0.0163	2.183	1852±74K

[a] Cold gas velocity : 8 cm/s (298K, 1atm); C/O: 0.6.

[b] T_{max} is the measured maximum flame temperature after radiation correction at H_p=1.0 cm.

Table S1.2. Summary of nonpremixed flame condition.

Nonpremixed flame	Flow rate		
	Toluene	C_2H_4	Air
	0.845ml/h	120SCCM	10 SLPM

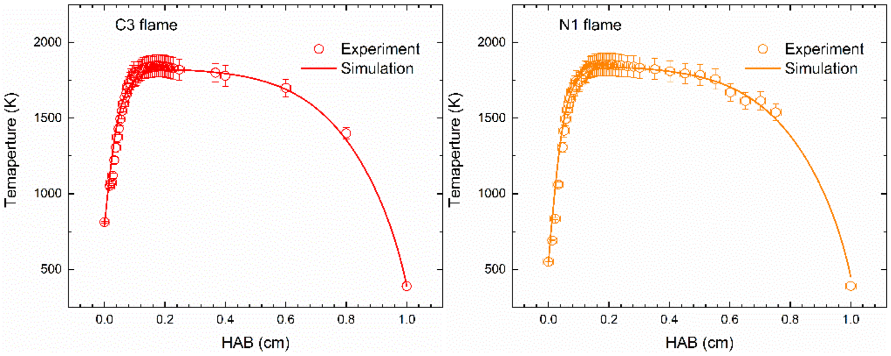

Figure S1.2. The temperature profiles of the premixed flames[3].

Supplementary Note 2: Additional MS information of matured soot particles.

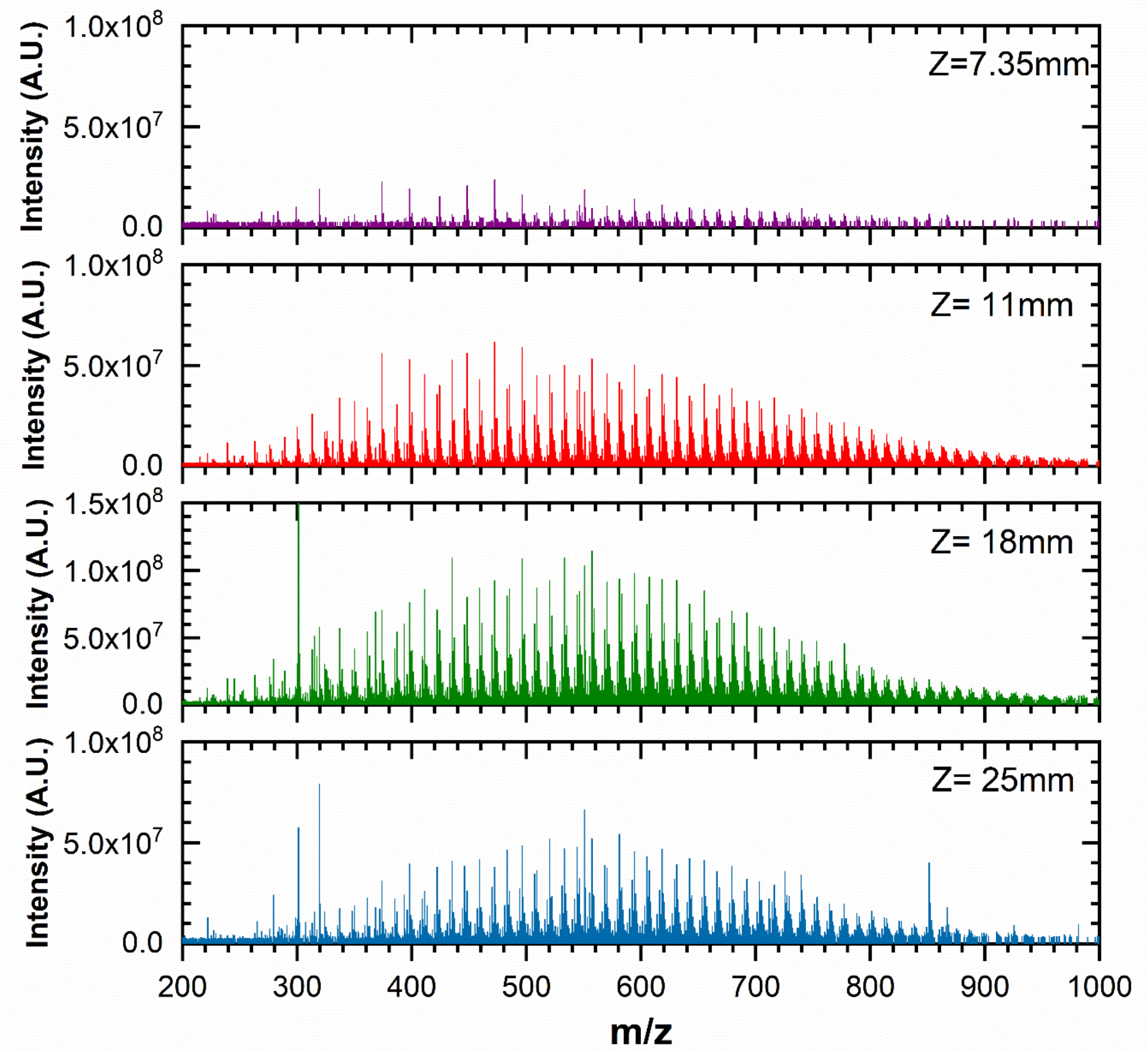

Figure S2.1.LDI/FT-ICR mass spectra of soot particles collected at the central line from different heights above the burner in a diffusion flame.

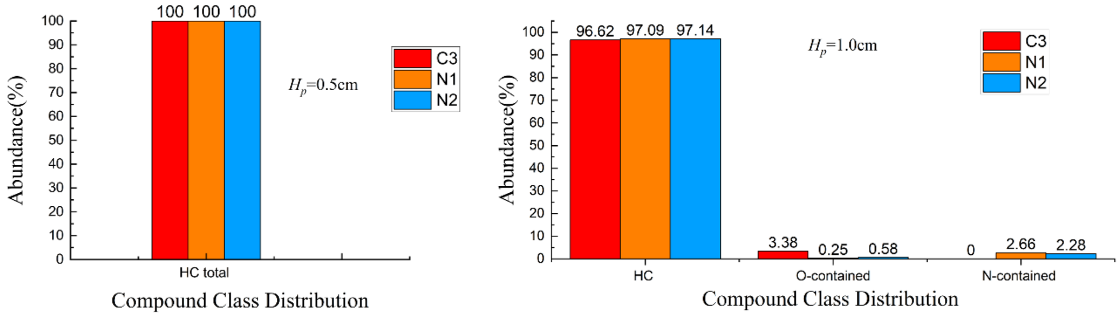

Figure S2.2. Compound class distribution of soot particle in premixed flames[3].

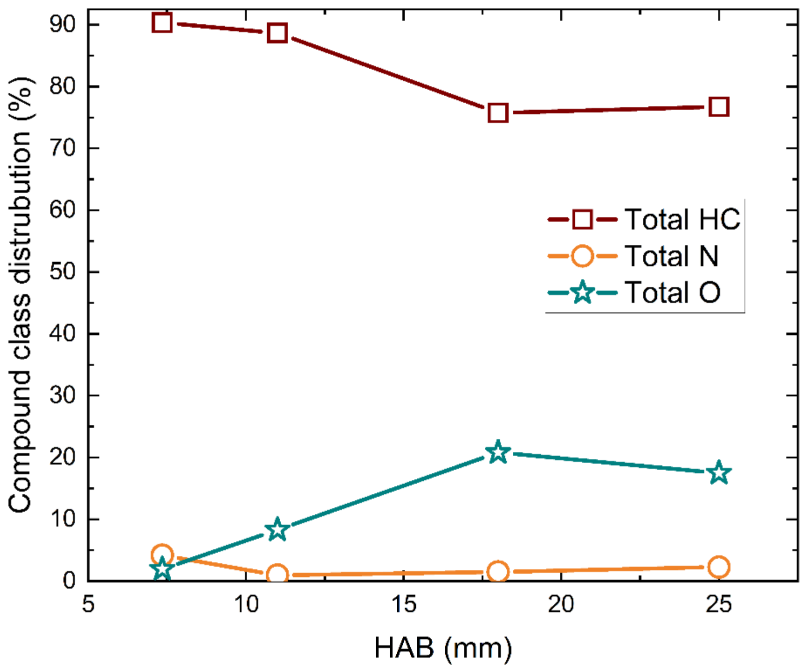

Figure S2.3. Compound class distribution of soot particle in a diffusion flame.

The distribution of elements in the particles at each height from the diffusion flame was directly derived from the Composer software, as illustrated in **Figure S2.2**. It is important to note that the figure indicates a nitrogen content of 3% in the NOC particles at a height of 7.35 mm. However, in practical scenarios, the low concentration of NOC, combined with the thermophoretic sampling method, results in a significantly reduced signal in the mass spectra. Consequently, the errors associated with elemental attribution are relatively large compared to more developed particles. To address this limitation, we focus exclusively on the major peaks that can be confidently attributed during the early stages of particle formation. Therefore, it is reasonable to assume that NOCs primarily consist of hydrocarbons.

Similarly, in premixed flames (as shown in **Figure S2.3**), it was observed that pure hydrocarbons were detected as NOCs. However, as the particles matured, nitrogen and oxygen-containing species were also detected.

In summary, during the inception process, NOCs were assumed to primarily consist of hydrocarbons. As the particles developed, nitrogen and oxygen-containing species became more prevalent.

Supplementary Note 3: The structures of PAHs predicted by various methods.

Table S3.1. The predicted structures of polycyclic aromatic hydrocarbons.

Molecular format	SNapS2	DFT or RMG	Stein- Fahr
C22H12	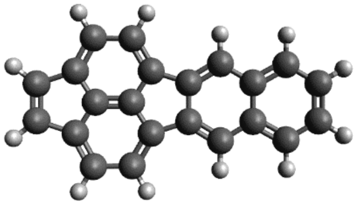	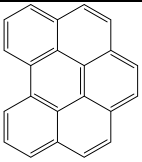	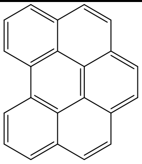
C24H12	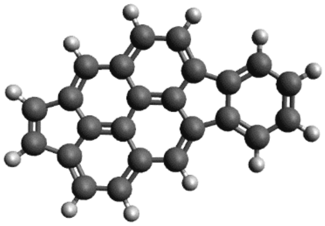	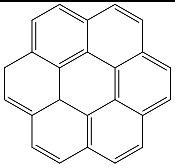	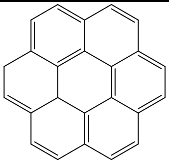
C28H14	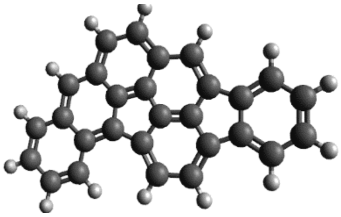	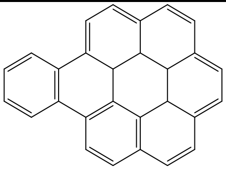	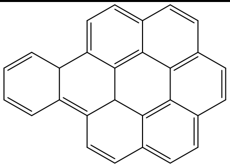
C30H14	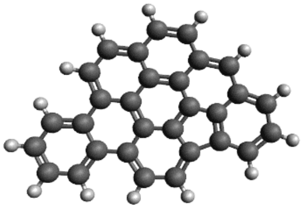	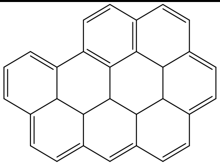	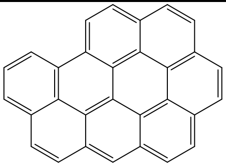
C32H14	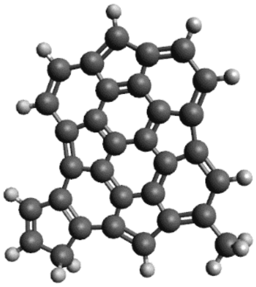	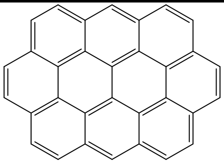	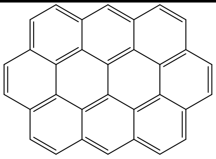

C34H16	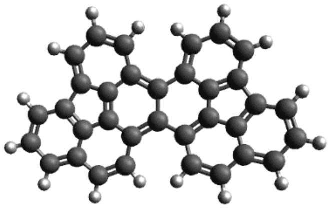	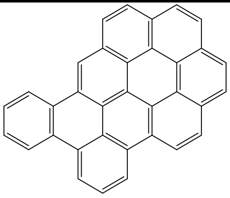	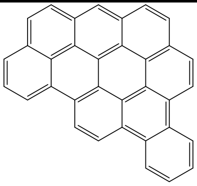
C36H16	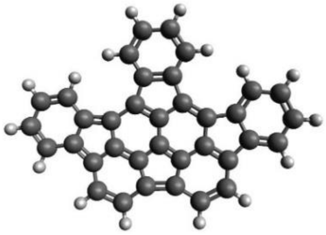	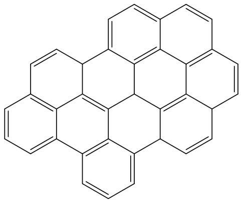	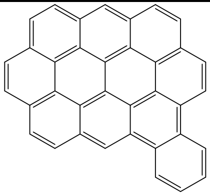
C38H16	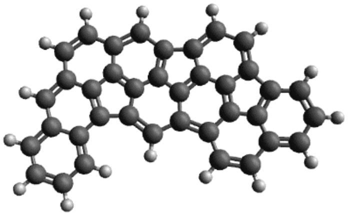	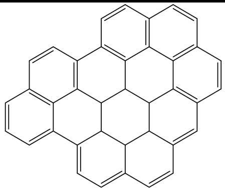	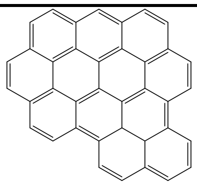
C40H16	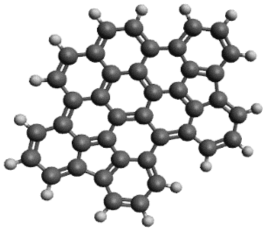	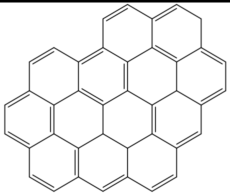	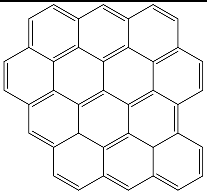
C42H16	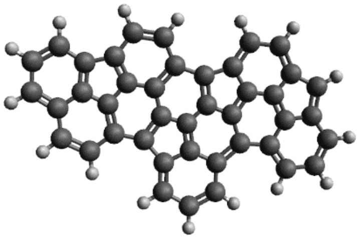	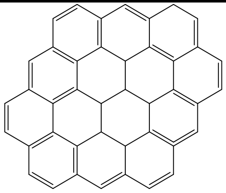	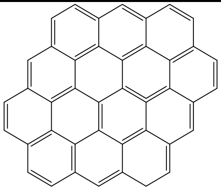

Supplementary Note 4: Theoretical Prediction of Thermochemistry

4.1 Quantum Chemical Calculation

In this study, all density functional theory (DFT) and composite compound method calculations were performed using the Gaussian 09 software package[4]. The M06-2X/6-311++G(d,p) method was used for the geometry optimizations, vibrational frequency calculations and hinder rotation treatments for lower frequency modes. All frequencies and zero point vibrational energies (ZPVEs) were scaled by 0.983 and 0.9698 respectively, which was recommended for the M06-2X functional by Zhao and Truhlar[5]. For the zero Kelvin energies (ZKEs) calculation part, compound methods: G3[6] was employed, which has been found to be quite reliable (arbitrarily, ~4 kJ mol^{-1} or 1 kcal mol^{-1}) for predicting the enthalpy of formation values for PAHs in a recent study from Liu et al.[7].

Atomization method were utilized to derive the enthalpies of formation at 0 K, in which, a molecule or radical is divided into its component atoms via the reaction:

$$C_xH_y \rightarrow x\ {}^3C + y\ {}^2H$$

in which the theoretical atomization energy at 0 K (TAE_0) can be calculated by:

$$TAE_0 = xH_0(\ {}^3C) + yH_0(\ {}^2H) - H_0(C_xH_y)$$

where H_0 is the enthalpy of formation at 0 K calculated using each compound method. Thereafter, the enthalpy of formation of the species ($\Delta_f H_0$) can be calculated knowing the TAE_0 and the standard formation enthalpies of the component atoms in their gaseous state from the ATcT[4-6], shown in Supplementary Table 4.1., via:

$$\Delta_f H_0(C_xH_y) = [x\Delta_f H_0(\ {}^3C) + y\Delta_f H_0(\ {}^2H)] - TAE_0$$

Table S4.1. Standard gaseous atomic formation enthalpies (kJ mol^{-1}).

T / K	C (^{3}P)	H ($^2S_{1/2}$)
0	711.38	216.034

The 298.15 K formation enthalpies, entropies and temperature-dependent heat capacities were then calculated from traditional statistical thermodynamics using the MultiWell program suite[8].

4.2 Group Additivity Method Calculation

The group additivity (GA) method was originally developed by Benson[9], and can be used to estimate the thermochemical properties of a molecule including the enthalpy of formation, entropy and heat capacities as function of temperature. In this study, the Reaction Mechanism Generator (RMG)[10,11,12] developed by in the William H. Green research group at MIT was utilized to perform the calculation.

Supplementary Note 5: The CFD simulation of nonpremixed flame.

5.1 Numerical model and computational configuration

A low-Mach number algorithm is employed in this study, which is similar to the implementation of a pimpleFoam solver built in OpenFOAM [13], with modifications done to ensure mass conservation for combustion related problems. The transport equations of momentum, species, and total enthalpy are solved. The gravity effect is considered in momentum equation. Chemical reactions are described with detailed thermochemical properties and reaction kinetics calculated using Cantera [14]. The optically thin approximation is used to calculate the radiation heat loss from triatomic gases and soot particles. These numerical models have been introduced and verified in ethylene coflow diffusion flames in previous studies [15,16,17].

A reduced PAH model were performed here for further simulation, which contained the reduced PAH model named sk99 [18] and the reduced toluene sub-mechanism [19]. It consists of 87 species and 577 reactions. This mechanism includes pathways for the formation of PAH species, which has the species up to pyrene. As shown in **Figure S5.1**, the mechanism has been verified for the ignition delay time of toluene and flame speed of ethylene and toluene mixture.

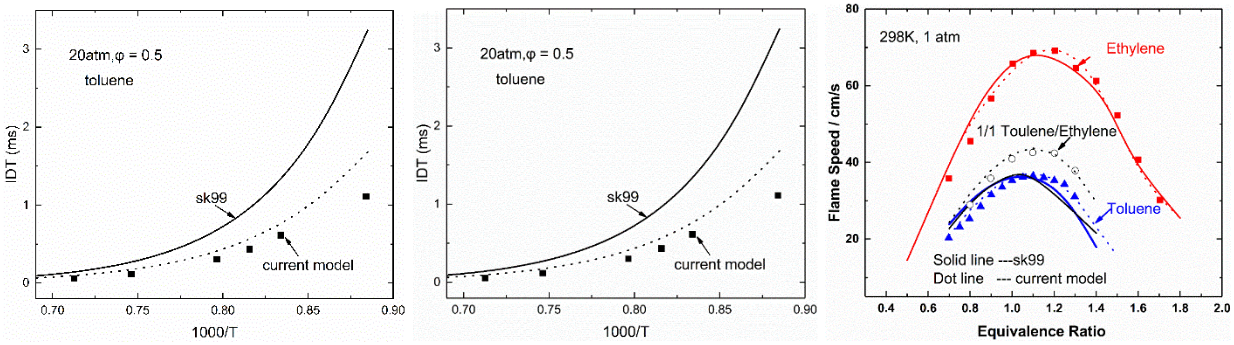

(a) (b) (c)

Figure S5.1. (a, b) Ignition delay time of toluene, (c) flame speed of mixture of ethylene and toluene.

Although the computational fluid dynamics (CFD) simulation only provides the temperature and species less than benzene for Monte Carlo simulations, it is still necessary to simulate the soot formation and oxidation, because the flame temperature is impacted by the radiation heat loss from soot particles. In this study, the hybrid method of moments (HMOM) is used to describe the soot aerosol dynamics. The processes of soot formation and oxidation include the nucleation, condensation, coagulation, surface growth, and oxidation. The nucleation process is based on the collision of pyrene molecules. The sticking coefficient of pyrene is 0.025 [20]. Soot surface growth is controlled by hydrogen abstraction carbon addition (HACA) mechanism and PAH dimer condensation. Reaction rates of HACA mechanism and soot oxidation are taken from Mueller [21] and the references therein. Soot oxidation by OH and O_2 is considered.

The two-dimensional computational domain used herein are shown in **Figure S5.2**. The domain extends radially outwards 50 mm and 80 mm downstream. The domain is also extended 10 mm upstream into the fuel and air tubes to better represent the inflow velocity distribution. Increasing the size of the domain further has no effect on the solution.

The computational domain is subdivided into 90 in the radial and 380 in the axial direction to form a structured, non-uniformly-spaced mesh with 33,960 cells.

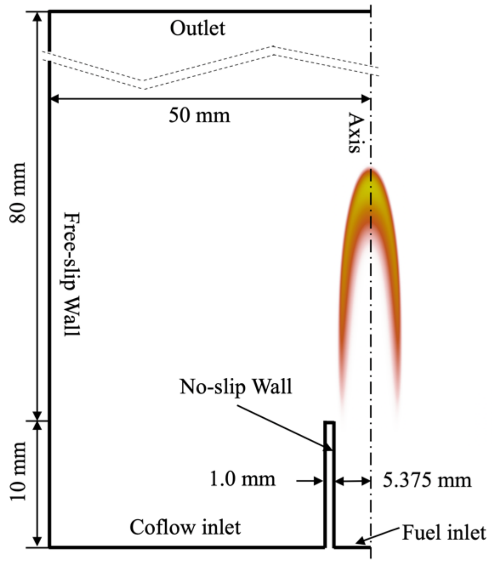

Figure S5.2. Computational domain with boundary conditions.

5.2 Simulation results

Figure S5.3 shows the measured and predicted temperature profiles in the flame centerline. The temperature is measured using a bare wire thermocouple. Overall, the predicted temperature agrees well with the measurement. In the region of height above burner less than 7 mm, the predicted temperature is lower than the measurement. This is may be attributed to the uncertainties of the measurement near the burner outlet, and the overestimated radiative heat loss by the optically thin radiation model.

The flue gas on the flame centerline is sampled by a ceramic tube with heated covering and then sent to the Gas Chromatography for gas composition analysis. **Figure S5.4** presents the comparison of measured and predicted gas species, including ethylene, acetylene, benzene, and naphthalene. Overall, the predicted gas species agree well with the measurement.

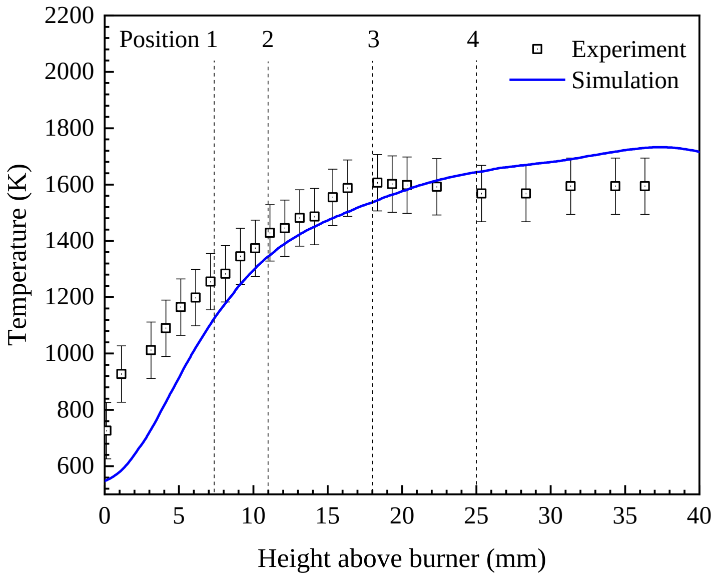

Figure S5.3. Measured and predicted temperature profiles in flame centerline.

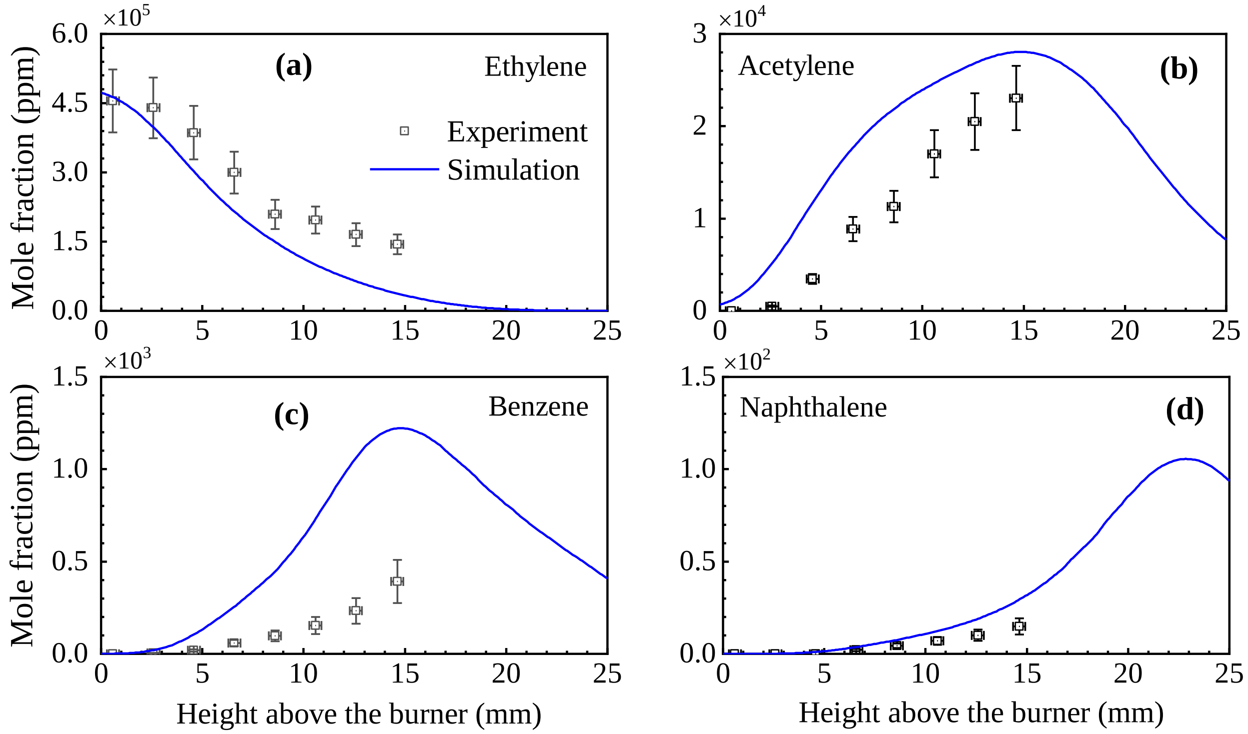

Figure S5.4. Measured and predicted gas species profiles in flame centerline.

Supplementary references

1. Camacho J. et al. Mobility size and mass of nascent soot particles in a benchmark premixed ethylene flame. *Combust. Flame* 2015, **162**(10)**:** 3810-3822.

2. Abid A. D., Camacho J., Sheen D. A.&Wang H. Quantitative measurement of soot particle size distribution in premixed flames-the burner-stabilized stagnation flame approach. *Combust. Flame* 2009, **156**(10)**:** 1862-1870.

3. Shao C. et al. Effects of ammonia addition on soot formation in ethylene laminar premixed flames. *Combust. Flame* 2022, **235:** 111698.

4. Foresman J., Ortiz J., Cioslowski J.&Fox D. Gaussian 09, Revision D. 01; Gaussian, Inc. *Wallingford, CT* 2009.

5. Zhao Y.&Truhlar D. G. The M06 suite of density functionals for main group thermochemistry, thermochemical kinetics, noncovalent interactions, excited states, and transition elements: two new functionals and systematic testing of four M06-class functionals and 12 other functionals. *Theor. Chem. Acc.* 2008, **120**(1)**:** 215-241.

6. Curtiss L. A., Raghavachari K., Redfern P. C., Rassolov V.&Pople J. A. Gaussian-3 (G3) theory for molecules containing first and second-row atoms. *The Journal of Chemical Physics* 1998, **109**(18)**:** 7764-7776.

7. Liu P., Li Y., Sarathy S. M.&Roberts W. L. Gas-to-Liquid Phase Transition of PAH at Flame Temperatures. *The Journal of Physical Chemistry A* 2020, **124**(19)**:** 3896-3903.

8. Barker J. R. et al. MultiWell-2016 Software Suite; J. R. Barker, University of Michigan, Ann Arbor, Michigan, USA. 2016.

9. Benson S. W. *Thermochemical kinetics: methods for the estimation of thermochemical data and rate parameters*. Wiley, 1968.

10. Gao C. W., Allen J. W., Green W. H.&West R. H. Reaction Mechanism Generator: Automatic construction of chemical kinetic mechanisms. *Computer Physics Communications* 2016, **203:** 212-225.

11. Magoon G. R.&Green W. H. Design and implementation of a next-generation software interface for on-the-fly quantum and force field calculations in automated reaction mechanism generation. *Comput. Chem. Eng.* 2013, **52:** 35-45.

12. Allen J. W., Goldsmith C. F.&Green W. H. Automatic estimation of pressure-dependent rate coefficients. *Physical Chemistry Chemical Physics* 2012, **14**(3)**:** 1131-1155.

13. Hassanaly M., Koo H., Lietz C. F., Chong S. T.&Raman V. A minimally-dissipative low-Mach number solver for complex reacting flows in OpenFOAM. *Comput Fluids* 2018, **162:** 11-25.

14. Goodwin D. G., Moffat H. K.&Speth R. L. *Cantera: An object-oriented software toolkit for chemical kinetics, thermodynamics, and transport processes, 2017, Version 2.3.0*: available at <http://www.cantera.org>.

15. Guo J., Selvaraj P., Tang Y., Im H. G.&Raman V. An analysis of soot formation pathways in laminar coflow ethylene flame at higher pressures. *AIAA Scitech 2020 Forum*; 2020. p. 1660.

16. Jin H. et al. Experimental and numerical study of polycyclic aromatic hydrocarbon formation in ethylene laminar co-flow diffusion flames. *Fuel* 2021, **289**.

17. Guo J., Tang Y., Raman V.&Im H. G. Numerical investigation of pressure effects on soot formation in laminar coflow ethylene/air diffusion flames. *Fuel* 2021.

18. Selvaraj P. et al. A computational study of ethylene–air sooting flames: Effects of large polycyclic aromatic hydrocarbons. *Combustion and Flame* 2016, **163:** 427-436.

19. Li Y. et al. Development of a reduced four-component (toluene/n-heptane/iso-octane/ethanol) gasoline surrogate model. *Fuel* 2019, **247:** 164-178.

20. Blanquart G.&Pitsch H. A joint volume-surface-hydrogen multi-variate model for soot formation. *Combustion generated fine carbonaceous particles* 2009**:** 437-463.

21. Mueller M. E. Large eddy simulation of soot evolution in turbulent reacting flows Stanford University, 2012.
